# Ultrafast near-infrared pyroelectric detector based on inhomogeneous plasmonic metasurface

**DOI:** 10.1038/s41377-024-01572-5

**Published:** 2024-09-06

**Authors:** Youyan Lu, Liyun Liu, Ruoqian Gao, Ying Xiong, Peiqing Sun, Zhanghao Wu, Kai Wu, Tong Yu, Kai Zhang, Cheng Zhang, Tarik Bourouina, Xiaofeng Li, Xiaoyi Liu

**Affiliations:** 1 School of Optoelectronic Science and Engineering & Collaborative Innovation Center of Suzhou Nano Science and Technology, Soochow University, Suzhou, 215006 China; 2 Key Lab of Advanced Optical Manufacturing Technologies of Jiangsu Province & Key Lab of Modern Optical Technologies of Education Ministry of China, Soochow University, Suzhou, 215006 China; 3 Suzhou Institute of Biomedical Engineering and Technology of the Chinese Academy of Sciences, Suzhou, 215163 China; 4 College of Intelligence Science and Technology, National University of Defense Technology, Changsha, 410073 China; 5 Laboratory of Science and Technology on Integrated Logistics Support, National University of Defense Technology, Changsha, 410073 China; 6 State Key Laboratory of Silicon and Advanced Semiconductor Materials, Zhejiang University, Hangzhou, 310027 China; 7 ESYCOM Lab, UMR 9007 CNRS, Univ Gustave Eiffel, 77454 Marne-la-Vallée, France; 8 CINTRA, IRL 3288 CNRS-NTU-THALES, Nanyang Technological University, Singapore, 637553 Singapore

**Keywords:** Nanophotonics and plasmonics, Optoelectronic devices and components

## Abstract

Pyroelectric (PE) detection technologies have attracted extensive attention due to the cooling-free, bias-free, and broadband properties. However, the PE signals are generated by the continuous energy conversion processes from light, heat, to electricity, normally leading to very slow response speeds. Herein, we design and fabricate a PE detector which shows extremely fast response in near-infrared (NIR) band by combining with the inhomogeneous plasmonic metasurface. The plasmonic effect dramatically accelerates the light-heat conversion process, unprecedentedly improving the NIR response speed by 2−4 orders of magnitude to 22 μs, faster than any reported infrared (IR) PE detector. We also innovatively introduce the concept of time resolution into the field of PE detection, which represents the detector’s ability to distinguish multiple fast-moving targets. Furthermore, the spatially inhomogeneous design overcomes the traditional narrowband constraint of plasmonic systems and thus ensures a wideband response from visible to NIR. This study provides a promising approach to develop next-generation IR PE detectors with ultrafast and broadband responses.

## Abstract (web summary)

We propose a plasmonic effect-assisted pyroelectric detector covered by an inhomogeneous Al metasurface, which exhibits ultrafast response speed and extended near-infrared response spectrum.

## Graphical abstract

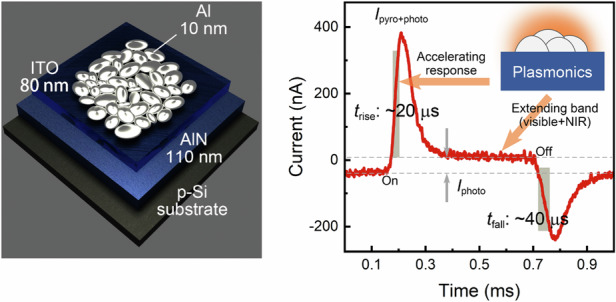

## Introduction

Infrared (IR) photodetection technologies have been paid widespread attention owing to their enormous potential in safety & security, military reconnaissance, medical testing, and industrial production^1–6^. The essence of photodetection is to convert the energy of incident light into an electrical signal through devices. According to different processes of energy conversion, common IR photodetectors can be roughly categorized into photon- and thermal-detection types^7^. The former usually consists of III-V group semiconductors and their compounds, typically being characterized by relatively fast response and high efficiency. However, the issues of complexity and high fabrication cost are long-standing; moreover, the cooling system is needed to prevent excessive heat during the excitation of carriers^8–10^.

Pyroelectric (PE) detectors, as a category of thermal detectors, possess a variety of advantages, including simple manufacturing, low cost, high stability, as well as no need for a cooling system and external bias in contrast to the photon-detection devices^11–14^. In particular, PE detectors exhibit an extremely wide working band since their photocurrent is generated by the variation of spontaneous polarization intensity induced by temperature fluctuations in PE materials, which is not restricted by the semiconductor bandgap. Consequently, they are capable of easily addressing various wavelengths of incident light, being quite applicable to serve as wide-spectral IR photodetectors. However, the generation of PE signals involves the energy consecutive conversion between light, heat and electricity^15,16^. As a result, the response time is relatively slow, typically on the order of tens of milliseconds, which is difficult to satisfy the application requirements such as real-time detection/imaging and dynamic target capture, etc.^17–21^

Over the past years, several initiatives aimed at optimizing the response speed of PE detectors have been reported. For instance, studies demonstrated that the response time can be reduced to tens of microseconds scale within a relatively wide waveband (~300−1000 nm) by leveraging zinc oxide (ZnO) nanostructured PE materials^22,23^; another study even declared that nano-picoseconds response times under pulsed light irradiation were achieved by virtue of combining metal plasmonic nanostructures and PE substrate^24^. Nevertheless, the wavebands of interest in most of the previous studies are either visible or in several but narrow bands. The former can be directly detected by mature commercial silicon (Si) detectors^25,26^, while the latter loses one of the most fundamental advantages of PE detectors, namely, broadband response. Additionally, the complex and costly manufacturing procedures are also difficult to avoid. Therefore, developing new schemes of IR PE detectors which can improve response speed and simultaneously maintain the desirable broadband detection, is still of very high importance.

Herein, we introduced non-continuous aluminum (Al) film as a metasurface layer to combine with the aluminum nitride (AlN) PE detectors. The entire construction of the proposed PE devices was realized by deposition approaches, without relying on the complex lithography and growth techniques. Metasurface components can excite plasmonic effect and accelerate the process of light-heat conversion, so as to improve the response speed. Meanwhile, the inhomogeneity of Al metasurface results in broadband plasmonic resonances. In this study, we experimentally achieved a wide working range covering visible and near-infrared (NIR) bands, and unprecedentedly shortened the NIR response time to 22 μs, which is improved by 2−4 orders of magnitude compared with traditional IR PE detectors. Moreover, we innovatively introduced the concept of time resolution into the field of PE detection, predicting that it is related to the incident pulse width and has a minimum limit. The effects of chopper frequency and bias voltage on the photodetection performance were also discussed to further uncover the physics behind the high performance. This study provides a promising way to develop low-cost next-generation IR PE detectors, which exhibit ultrafast and broadband PE responses.

## Results

### Operating principle

**Fig. 1:**
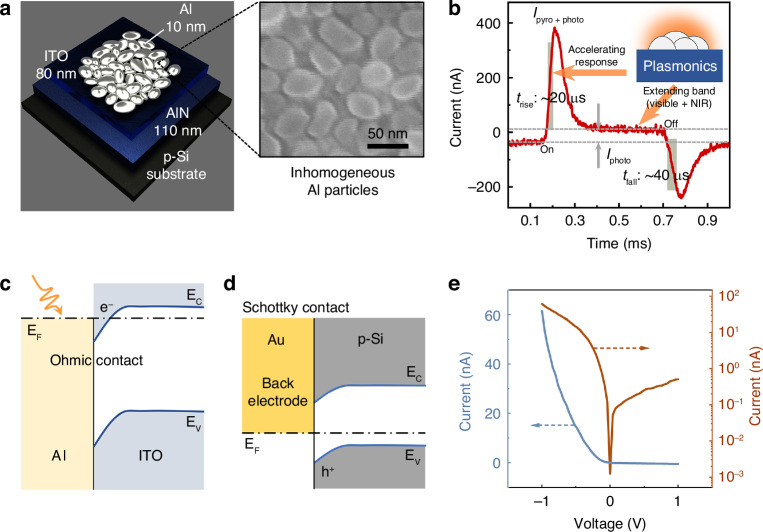
Operating principle of the proposed PE detector. **a** Structure diagram of the proposed PE detector. Top-view SEM image of the Al particle metasurface is also given. **b** Typical measured photocurrent waveform. The inset shows the schematic of the plasmonic effect excited by Al particles. **c** Energy band state at Al-ITO interface. **d** Energy band state at Au (back electrode)-Si interface. **e** I-V characteristics of the proposed PE detector, respectively depicted in decimal (left) and logarithmic (right) coordinate system

Figure 1a presents the structure schematic of the proposed AlN PE detectors. A 110-nm-thick AlN layer and an 80-nm-thick indium tin oxide (ITO) layer were successively deposited on a p-type Si substrate, respectively acting as the PE material and the electrode. Then, a 10-nm-thick Al film was deposited at the top of the detector. Due to its ultrathin thickness, the Al film would spontaneously form a non-continuous and inhomogeneous particle metasurface during deposition. The scanning electron microscope (SEM) image in Fig. 1a exhibits the morphology and distribution of top Al particles. The macroscopic/microscopic images of the prepared detector (see Fig. S1, Supplementary Information) are also given. Detailed preparation procedures are described in the Methods section.

Based on the unique Al particle metasurface structure, the proposed PE detector possesses valuable characteristics of ultrafast and broadband response. Figure 1b presents a typical measured photocurrent waveform, in which the signal is comprised of PE current (*I*_pyro_) and photovoltaic (PV) current (*I*_photo_)^27,28^. On the one hand, the PE signal *I*_pyro_ is determined by the temperature variation rate of the AlN layer. As an inherent ability of PE materials, the spontaneous polarization status of the AlN layer can be modulated by temperature variation. When the temperature increases, the positive and negative charges in the material lattice will spatially separate, consequently leading to the PE potential and current^29,30^. The operating principles of conventional PE detectors are based on this phenomenon, which contains the energy conversions between light, heat, and electricity, and thus their response speed is severely affected. In contrast, the energy of incident light in this work is firstly converted into localized surface plasmon resonances (LSPRs) excited by Al particles. These LSPRs subsequently occur radiative decay and transport heat towards the AlN layer at femto-to-picosecond scales, thereby enabling the response speed to be effectively accelerated^31,32^. The photocurrent signal in Fig. 1b exhibits a rise time (*t*_rise_, defined as the time from 10 to 90% of maximum peak) of ~20 µs and a fall time (*t*_fall_, defined as the time from 90 to 10% of maximum peak) of ~40 µs, which are significantly faster than any IR PE detector reported before^16,17,19^; on the other hand, the hot electrons excited by the non-radiative decay of LSPRs, cross the underlying ITO/AlN layers and directly form the PV signal *I*_photo_. As known, the plasmonic resonant frequency generally depends on the shape and size of the nanostructures^33–35^. In this study, we have experimentally demonstrated that the working band of such a PE detector extends from visible to NIR, which should be ascribed to the multiple plasmonic resonances excited by the top inhomogeneous Al particles. The bandgap of AlN is 6.2 eV, even larger than that of Si. Therefore, the *I*_photo_ in NIR can only be generated by LSPRs rather than the PV effect of materials^31,36^. The schematic of the signal generation in such a detector is explained in Fig. S2, Supplementary Information. The roles of LSPRs in modulating the response speed and bandwidth will be carefully discussed later.

Figures 1c, d illustrate the energy band states at two metal-semiconductor interfaces of this detector. The top Al particles of the detector form an ohmic contact with the n-type ITO layer below, allowing the hot electrons excited by LSPRs to freely pass through the interface. Additionally, a Schottky contact is formed between the Si substrate and the gold (Au) back electrode during testing. This Schottky junction can create a space charge region, which is necessary for PE detectors to be able to work at zero bias^22,37^. The detailed energy band diagram of such a structure is expressed in Fig. S3, Supplementary Information. The I-V characteristics, that are respectively depicted in decimal and logarithmic coordinate systems in Fig. 1e, indicate that the designed detector has excellent rectification properties. The presence of the Schottky junction formed by Au-Si contact has also been confirmed.

### LSPRs-based response speed acceleration

**Fig. 2:**
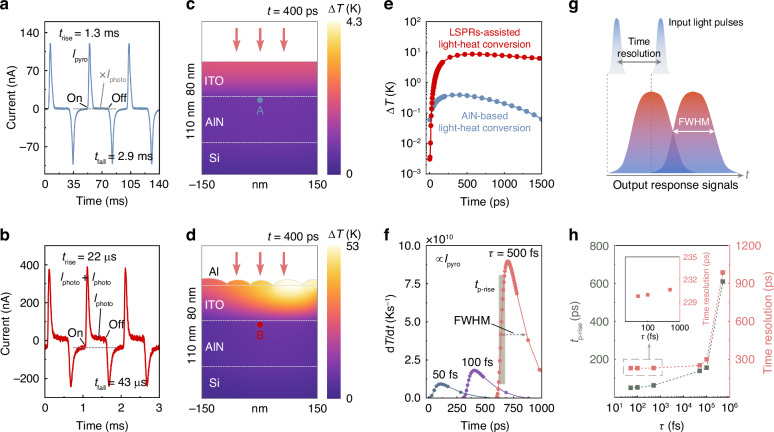
LSPRs-induced response speed acceleration. **a** Measured response waveform of the control sample without plasmonic metasurface. **b** Measured response waveform of the proposed detector with plasmonic metasurface. **c** Simulated distribution of temperature variation for the control sample. **d** Simulated distribution of temperature variation for the proposed detector with plasmonic metasurface. **e** Simulated ∆*T* profiles of the AlN layers in two detectors. **f** Simulated d*T*/d*t* of the AlN layer in the proposed detector under different incident light pulses. **g** Diagram of the concept of time resolution for PE detectors. **h** Dependences of *t*_p-rise_ and time resolution on the incident pulse width *τ*

In order to further explain the accelerating mechanisms of LSPRs on response speed, an AlN PE detector sample without Al metasurface was prepared as a control. Figure 2a, b present the measured photocurrent waveforms of AlN PE detectors without and with Al particle metasurface. The measurements were conducted at an incident light wavelength of 1110 nm with zero bias. The upper limits of the operating frequency of choppers are determined by the response speed, and the choppers’ frequencies were adopted at 1000 and 21 Hz in these two cases. For the control sample, the PE current *I*_pyro_ is mainly related to the rate of temperature change of AlN film (d*T*/d*t*)^13^:

1 $${I}_{\text{pyro}}(t)={pS}\frac{\text{d}T(t)}{\text{d}t}$$

where *p* is the PE coefficient and *S* represents the light-sensitive area of the detector. The positive and negative peaks in the curve thus correspond to the processes of temperature rise (from light-on) and temperature fall (from light-off). The relatively long *t*_rise_ and *t*_fall_, respectively, up to 1.3 and 2.9 ms, indicate slow temperature variation of the AlN layer. Compared with the control sample, the detector with Al metasurface introduces a plasmonic effect to assist the energy conversion between light and heat. The thermal energy generated by the radiative decay of LSPRs then crosses the ITO film and transports towards the AlN layer. The entire process only takes tens of picoseconds. As a result, the *t*_rise_ and *t*_fall_ of this detector are 22 and 43 µs, which are two orders of magnitude faster than that of the control sample. The presence of PV current *I*_photo_ confirms that the LSPRs indeed participate in the energy conversion process described above. The wavelengths used in measurements exceed the bands of intrinsic absorption of Si and AlN, hence *I*_photo_ could only be formed by the hot electrons excited by non-radiative decay of LSPRs. This part of the signal is absent in the case of the control sample. In contrast, it exists throughout the period of light irradiation in the LSPRs-assisted detector, even if the temperature stabilizes and the PE signal subsides.

A series of simulations based on the finite element method have also been performed to theoretically analyze the process of light-heat conversion in the detectors. Figure 2c, d demonstrate the distributions of temperature variation after a 200 fs light pulse irradiation in two detectors, intuitively exhibiting the difference in light-heat conversion processes without and with the assistance of LSPRs. The time of these two simulations selected is 400 ps. Obviously, the temperature variation assisted by LSPRs is much more dramatic, and faster than another one. The simulation results at 100, 200, and 300 ps are also provided in Fig. S4, Supplementary Information. Figure 2e aggregates the simulated temperature variation trends of the AlN layers in two detectors (the detailed locations of temperature sampling are points A and B, marked in Fig. 2c, d). Here ∆*T* is defined by ∆*T* = *T*(*t*)−*T*_0_, where *T*_0_ is the initial temperature. It displays that the heat generated by LSPRs takes only tens to hundreds of picoseconds to transfer inside the device. Moreover, since *I*_pyro_ is proportional to d*T*/d*t* according to Eq. 1, the response time obtained under pulsed light can be derived by the temperature variation rate. Figure 2f shows the simulated d*T*/d*t* of the AlN layer induced by different incident light pulses, with the pulse widths *t* = 50, 100, and 500 fs. The simulation results under 50, 100, and 500 ps light pulses are also given in Fig. S5, Supplementary Information. Apparently, this kind of response time, which is named *t*_p-rise_, is largely decided by the width of the light pulse, and is naturally much faster than that measured by continuous light^24,38^. Therefore, the response speeds obtained under continuous and pulsed light should be distinguished^39^.

Compared with *t*_p-rise_, the full width at half maximum (FWHM) of such pulse-induced signals is more worth discussing. As can be seen from Fig. 2g, if two neighboring pulses are temporally too close, their response signals may not be distinguishable. The permitted minimum time interval between two pulses can be approximately equal to the FWHM of response signals. It can be regarded as representing the time resolution of the detector, which is of great significance in practice, e.g., as an indicator for assessing the detector’s ability to recognize multiple fast-moving targets. Figure 2h portrays the dependences of *t*_p-rise_ and time resolution on the incident pulse width *τ*. Overall, both these two parameters decrease as *τ* decreases. However, when *τ* decreases below the order of picoseconds, the time resolution tends to converge to a minimum value of ~230 ps. Theoretically, it is the ability limit of this detector to distinguish between two temporally neighboring incident pulses, which is intrinsically restricted by the material and structure of the device. Remarkably, the predicted minimum time resolution (~230 ps) of the proposed PE detector is already close to the state-of-the-art reported in the previous literature^24^. The results of the above simulations validate the enormous potential for realizing ultrafast, high-time resolution IR photodetection in PE detectors by virtue of constructing plasmonic metasurfaces.

### Broadband response induced by inhomogeneous metasurface

**Fig. 3:**
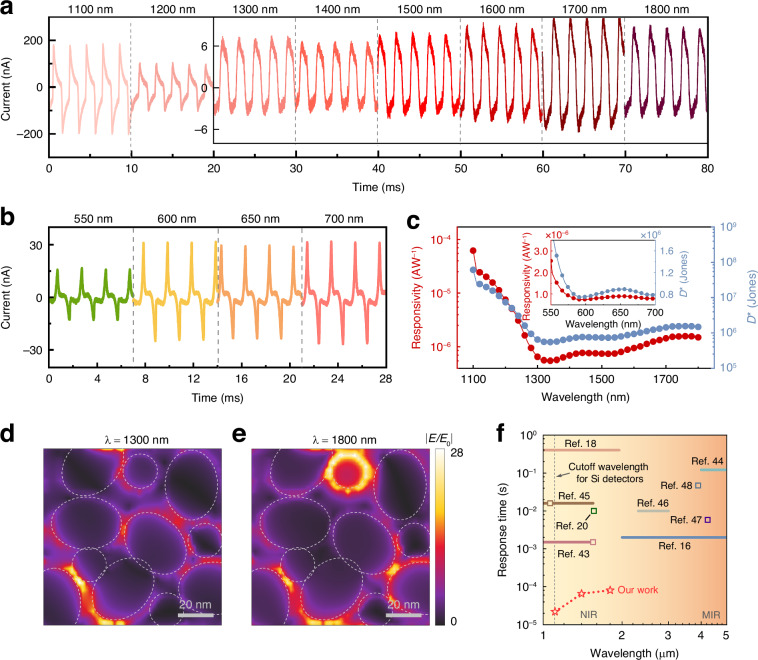
Broadband response based on inhomogeneous metasurface. **a** Measured waveforms of the proposed PE detector from 1100−1800 nm. **b** Measured waveforms of the proposed PE detector from 550−700 nm. **c** Responsivity *R* and specific detectivity D* of the proposed PE detector in 550−700 nm and 1100−1800 nm ranges. Electric field intensity distributions at the interface between inhomogeneous Al particles and ITO layer are given, with the incident light wavelengths of **d** 1300 nm and **e** 1800 nm. The color bar indicates the normalized electric field intensity. **f** Comparison of our study with other literature with regard to the response time and bandwidth

The plasmonic effect has an obvious structural dependence, and regular plasmonic structures usually lead to narrowband resonant frequencies. However, the inhomogeneous Al particle metasurface in our work provides possibilities to realize a wide resonant band covering visible and NIR regions. We continuously investigated the response characteristics of the proposed PE detector at different incident wavelengths. The waveforms in NIR (1100−1800 nm) and visible (550−700 nm) ranges measured with the conditions of zero bias and 500 Hz chopper frequency are respectively exhibited in Fig. 3a, b. The switching characteristic profiles of the detector over a wide spectrum are also given (see Fig. S6, Supplementary Information). Obviously, the proposed detector demonstrates remarkable broadband detection capability, and the waveform is quite distinguishable even at 1800 nm. In view of the structure-dependent properties of LSPRs, it is rare to practicably increase the response speed while still maintaining the original advantage of broadband detection in IR PE detectors. Fortunately, the designed detector with spatially inhomogeneous plasmonic metasurface offers a feasible approach to achieve this target.

In order to evaluate the capability of photoelectric conversion of designed detector over broadband range, the photoresponsivity *R* in NIR (1100−1800 nm) and visible (550−700 nm) regions are calculated according to the experimental results, as shown by the red curves in Fig. 3c. It can be defined as *R* = (*I*_light_−*I*_dark_)/*P*, where *I*_light_ is the total current generated by light irradiation, and *I*_dark_ is the dark current^40^. *P* is the power of incident light (see Fig. S7a, Supplementary Information). The responsivity *R* can also be expressed in terms of external quantum efficiency^41^, which is carefully described in the content corresponding to Fig. S7b, Supplementary Information. Due to the assistance of LSPRs, this detector exhibits considerable photodetection efficiency within the wide spectrum, whose responsivity in NIR is even larger than that in the visible region. At 1110 nm, which is beyond the working band of commercial Si detectors, the measured responsivity is close to 3 × 10^−5^ AW^−1^, and it still maintains 1.5 × 10^−6^ AW^−1^ at 1800 nm. On the other hand, the specific detectivity D* of the proposed detector is also explored (see blue curves in Fig. 3c). It is an important parameter to examine the theoretical detection capability of the detector. When the dark current is considered the dominant noise, the specific detectivity can be characterized as follows^42^:

2 $${\text{D}}^{* }=\frac{\sqrt{\text{S}}}{\sqrt{2\text{q}{\text{I}}_{\text{dark}}}}\text{R}$$

It can be observed that the D* of the detector is as high as 2.68 × 10^7^ Jones at 1110 nm, and still remains 1.48 × 10^6^ Jones at 1800 nm. Note that the wavebands adopted in the experiments above were determined by the working range of the photoelectric test system, which are not the limit of bandwidth of the proposed detector. Both the responsivity and the specific detectivity illustrate that such LSPRs-assisted PE detector has excellent photodetection capability over broadband range.

By virtue of the time-domain finite-difference method, the schematics of simulated electric field intensity distributions at the interface between inhomogeneous Al particles and the ITO layer are obtained (see Fig. 3d, e). The incident light wavelengths are respectively 1300 and 1800 nm. As mentioned before, the excitation of LSPRs has a strong structural dependence. Owing to the randomness of Al particles, multiple localized plasmonic resonances with various electric field confinement locations would be excited corresponding to different incident light wavelengths. Accordingly, the wide working band of such detector should be dominantly ascribed to the inhomogeneity of Al metasurface, which enables the advantage of broadband response for PE detectors to be maintained to some extent when improving their speed through exciting LSPRs.

Figure 3f depicts the response speeds and bandwidths of the proposed detector, where some representative PE detectors reported previously are included for comparison^16,18,20,43–48^. The coordinates of the horizontal and vertical axes corresponding to the horizontal bars, respectively, represent the working bands and response times of the detectors, while the hollow frames represent the concrete wavelengths they utilized to measure the response times. As can be seen, the response speeds of conventional IR PE detectors are generally slow, and some of them merely possess narrow detection bands. Our work, however, balances the response speed and bandwidth, and dramatically improves the response speed from milliseconds to microseconds regime. In particular, in the NIR region beyond 1100 nm (the cutoff wavelength for commercial Si detectors), the speed of the designed PE detector is faster than any IR PE detector reported before. Figure S8, Supplementary Information, depicts the detailed response times of the detector at each wavelength, demonstrating not only the generalized response speed improvements in broadband NIR range, but also the good stability of the designed device in repeated measurements. It offers a great significance for exploiting novel PE detectors with excellent photodetection performance.

### Effects of chopper frequency and bias voltage

**Fig. 4:**
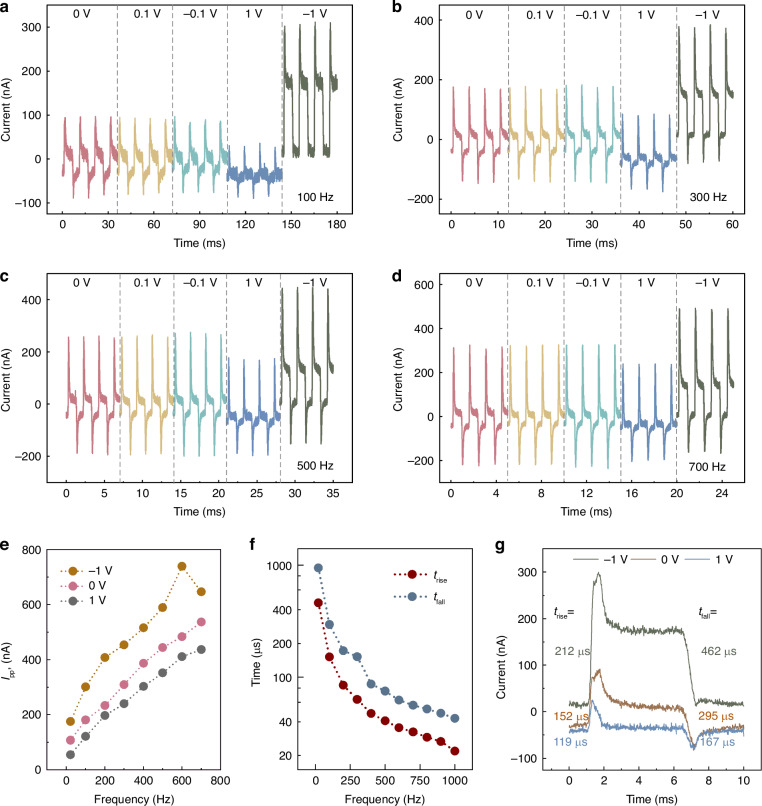
Effects of chopper frequency and bias voltage on the photodetection performance. Measured response waveforms of the proposed PE detector under different bias voltages, with the chopper frequencies of **a** 100 Hz, **b** 300 Hz, **c** 500 Hz, and **d** 700 Hz. **e** Dependence between *I*_pp’_ and chopper frequency with the bias voltages of 0 and ±1 V. **f** Rise and fall times of response signal with different chopper frequencies. **g** Waveforms extracted from Fig. 4a with the bias voltages of 0 and ±1 V

The frequency of the chopper and bias voltage adopted in experiments would dramatically affect the measurement results. Figure 4a−d display the different waveforms of the proposed PE detector obtained by varying the bias voltages (0, ±0.1, ±1 V) and optical chopper frequencies (100, 300, 500, 700 Hz). The wavelength of incident light is 1110 nm. The waveforms obtained with other chopper frequencies (21, 200, 400, 600 Hz) are given in Fig. S9, Supplementary Information. From the waveforms above, we extracted the peak-to-peak current *I*_pp’_, which refers to the difference between the instantaneous peak output currents corresponding to the moments of incident light-on and light-off, to represent the intensity of the response signal. The dependence between *I*_pp’_ and chopper frequencies with different bias voltages was then plotted as Fig. 4e. Firstly, it can be observed that at the same bias voltage, the *I*_pp’_ generally increases with increasing the chopper frequency. For instance, at zero bias, the *I*_pp’_ is only 107 nA with the chopper frequency of 21 Hz, but reaches 537 nA when the chopper frequency becomes 700 Hz. Secondly, at the same chopper frequency, the values of *I*_pp’_ under negative bias are significantly higher than those under positive bias. This is because when a positive bias is applied, the width of the depletion zone at the interface between Au and Si decreases, making the built-in electric field and corresponding *I*_photo_ very weak. Additionally, the rise of background temperature induced by large bias will also attenuate the PE effect and diminish the *I*_pyro_^49^. This effect is noticeable against a background of small *I*_photo_ under positive bias, but not sufficient to offset the increase of *I*_photo_ under negative bias. This conclusion is also consistent with the trend of responsivity *R* obtained over the wide waveband with different bias voltages (see Fig. S10, Supplementary Information), as well as the I-V characteristics in Fig. 1e.

In addition, the rise and fall times in waveforms at zero bias are extracted to analyze the dependence between response speed and chopper frequency varying from 21 to 1000 Hz in Fig. 4f, where the waveforms corresponding to 800, 900, and 1000 Hz are supplemented in Fig. S11, Supplementary Information. Apparently, as the chopper frequency increases, the response speed of the detector improves rapidly. The rise and fall times corresponding to 1000 Hz are nearly 20 times faster than those corresponding to 21 Hz. It is because that the larger chopper frequency represents the shorter interval between incident light-on and light-off, enabling the temperature variation rate and response speed, to be accelerated. The response intensity, as mentioned before, will be subsequently enhanced as well. This conclusion is consistent with others reported by literature, but the speed of our proposed detector is faster^43^. Note that the chopper frequency cannot be increased indefinitely, otherwise the temperature variation rate will be unable to keep pace with it and make the waveform distorted. It is worth mentioning that the rise time at a certain chopper frequency is always shorter than the corresponding fall time, which further illustrates the contribution that combining PE and PV effects to the responsivity enhancement^50^.

Finally, the effects of applied bias voltage on the response speed of the detector are discussed. The individual waveforms with the bias voltages of 0 and ±1 V are extracted from Fig. 4a, and are redrawn in Fig. 4g. The rise and fall times with positive bias are shorter than those with negative bias, which can be explained by the change in the width of the depletion region when bias is applied. To summarize, the positive bias improves the response speed but weakens the intensity of the response signal, while the negative bias does the opposite.

## Discussion

In conclusion, we demonstrate a scheme for designing IR PE detectors with ultrafast detection ability by virtue of an inhomogeneous plasmonic metasurface. With the assistance of LSPRs excited by the Al particle metasurface, the energy conversion process between light and heat is effectively accelerated. As a result, the response speed of the proposed IR device has been unprecedentedly improved by two to four orders of magnitude, with rise and fall times of 22 and 43 μs, faster than any IR PE detector reported previously. Meanwhile, the concept of time resolution is innovatively introduced into the field of PE detection, and the minimum time resolution of the proposed detector is predicted to be ~230 ps, indicating a superior potential capability of distinguishing multiple fast-moving targets. Moreover, the inhomogeneous Al particles can excite multiple LSPRs at different wavelengths, leading to a wide working band covering visible (550−700 nm) and NIR (1100−1800 nm) ranges. The responsivity and specific detectivity over a wide spectrum are also calculated to validate its broadband detection capability. It can be concluded that the proposed PE detector truly achieves the balance between response speed and bandwidth. In particular, in the NIR region beyond the working band of commercial Si detectors, the response speed of this detector has made remarkable progress compared with other studies. Finally, the effects of bias voltage and optical chopper frequency on the detection performance are systematically analyzed. It is worth mentioning that the entire preparation process of this device can be easily completed by the deposition method, which is beneficial to cost control. This study offers a promising way to develop next-generation IR PE detectors with excellent photodetection performance as well as low cost, being expected to be extensively applied in both military and civilian fields.

## Materials and methods

### Device fabrication

A boron-doped Si wafer (111-type) with a resistivity of 1−20 Ωcm was employed as the substrate of PE detectors. A 110-nm-thick AlN film was then prepared on the substrate by simultaneously depositing Al through the magnetron sputtering method and infusing nitrogen (N_2_) gas with a high surrounding temperature. The ratio of the gas flow rate of argon (Ar) and N_2_ was 1:5, and the substrate temperature was 200 °C. This yielded a polycrystalline AlN film possessing c-axis orientation and nanoscale surface roughness. Subsequently, an 80-nm-thick ITO film and a 10-nm-thick non-continuous Al film were successively deposited on the top of the detector. The Al film would spontaneously shrink into inhomogeneous particles owing to its ultrathin thickness. If the diameters of photosensitive areas of the detectors are larger than 0.5 mm, they can be simply defined by a patterned mask plate during the deposition process; otherwise, the ultraviolet (UV) lithography and inductively coupled plasma reactive ion etching (ICP-RIE) techniques are needed to define the small photosensitive areas. Finally, an approximately 70-nm-thick Au film was deposited on the back of the detector to act as the back electrode.

### Characterization and measurements

The morphology of the top Al particles is characterized by scanning electron microscopy (Zeiss, sigma 300). For the measurements of photodetection performance, a supercontinuum laser (NKT Photonics) was adopted as the light source, which was then modulated by an acousto-optic tunable filter to achieve monochromatic output light. The response waveforms, photocurrents, and I-V characteristic curves in this study were measured and recorded by a micro-area optoelectronic test system (META). It consists of a signal generator, an optical chopper, a preamplifier (SR570), a lock-in amplifier (SR830), and a digital source meter (Keithley 2635). The light spot was focused on the photosensitive area of the detector, and an optical power meter from Thorlabs (S148C) was used to measure the intensity of the transmission signal.

### Simulations

A series of simulations were constructed by the finite element method to calculate the temperature variation process in the proposed PE detector. A Gaussian laser with a power density of 3 W∙cm^−2^ and a tunable pulse width was used as the irradiation source. The absorption characteristics of the detector were first analyzed in the frequency domain of the wave optics module, and the absorptivity was calculated by integrating the absorbed power density, i.e., the plasmonic resonance loss, over the volume. Secondly, the absorbed energy was introduced into the solid-state heat transfer module to investigate the transient temperature changes on the AlN PE layer of the detector. Additionally, some simulations based on the finite-difference time-domain method were also completed to investigate the electric field intensity distribution on the top Al metasurface when LSPRs were excited. A plane wave was adopted as the normal incidence source, with wavelengths of 1300 and 1800 nm. The modeling of the Al particle metasurface is random, to some extent, so as to approach its practical morphology as closely as possible.

## Data and materials availability

The authors declare that the data supporting the findings of this study are available with the paper and its Supplementary Information files. The data that support the findings of this study are available from the corresponding author upon reasonable request.

## Supplementary information

Supplementary Information
