# Supplementary material for: Ultrafast near-infrared pyroelectric detector based on inhomogeneous plasmonic metasurface
Source: Light Sci Appl. 2024 Sep 6;13:241. doi: 10.1038/s41377-024-01572-5 (PMC11377428; doi:10.1038/s41377-024-01572-5)
Supplement: Supplementary file 1 — Supplementary Information [file 41377_2024_1572_MOESM1_ESM.docx]

Supplementary Information for

Ultrafast near-infrared pyroelectric detector based on inhomogeneous plasmonic metasurface

Youyan Lu^1,2^, Liyun Liu^1,2^, Ruoqian Gao^3^, Ying Xiong^4,5^, Peiqing Sun^1,2^, Zhanghao Wu^1,2^, Kai Wu^1,2^, Tong Yu^1,2^, Kai Zhang^1,2^, Cheng Zhang^1,2,6,*^, Tarik Bourouina^7,8^, Xiaofeng Li^1,2,*^, Xiaoyi Liu^1,2,*^

^1^School of Optoelectronic Science and Engineering & Collaborative Innovation Center of Suzhou Nano Science and Technology, Soochow University, Suzhou 215006, China;

^2^Key Lab of Advanced Optical Manufacturing Technologies of Jiangsu Province & Key Lab of Modern Optical Technologies of Education Ministry of China, Soochow University, Suzhou 215006, China;

^3^Suzhou Institute of Biomedical Engineering and Technology of the Chinese Academy of Sciences, Suzhou 215163, China;

^4^College of Intelligence Science and Technology, National University of Defense Technology, Changsha 410073, China;

^5^Laboratory of Science and Technology on Integrated Logistics Support, National University of Defense Technology, Changsha 410073, China;

^6^State Key Laboratory of Silicon and Advanced Semiconductor Materials, Zhejiang University, Hangzhou 310027, China;

^7^ESYCOM Lab, UMR 9007 CNRS, Univ Gustave Eiffel, 77454 Marne-la-Vallée, France;

^8^CINTRA, IRL 3288 CNRS-NTU-THALES, Nanyang Technological University, 637553, Singapore.

^*^Corresponding email: zhangc@suda.edu.cn; [xfli@suda.edu.cn](mailto:xfli@suda.edu.cn); xyliu@suda.edu.cn


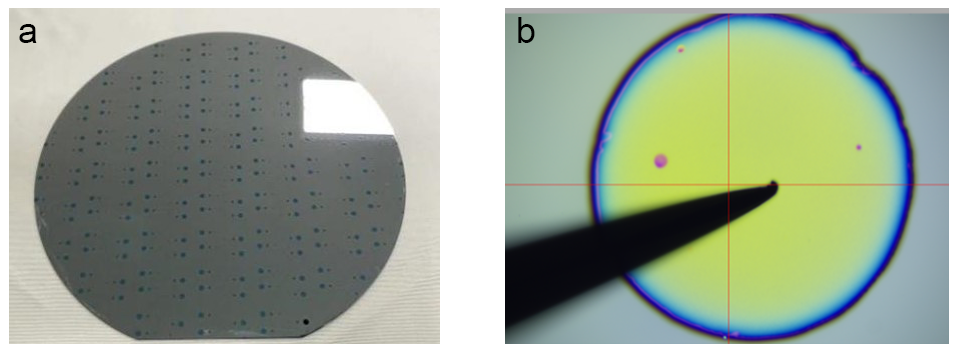


**Fig. S1 Images of the proposed pyroelectric detector.** **a** Macroscopic image of the detectors prepared on a Si wafer. **b** Microscopic image of the photosensitive area of the detector.


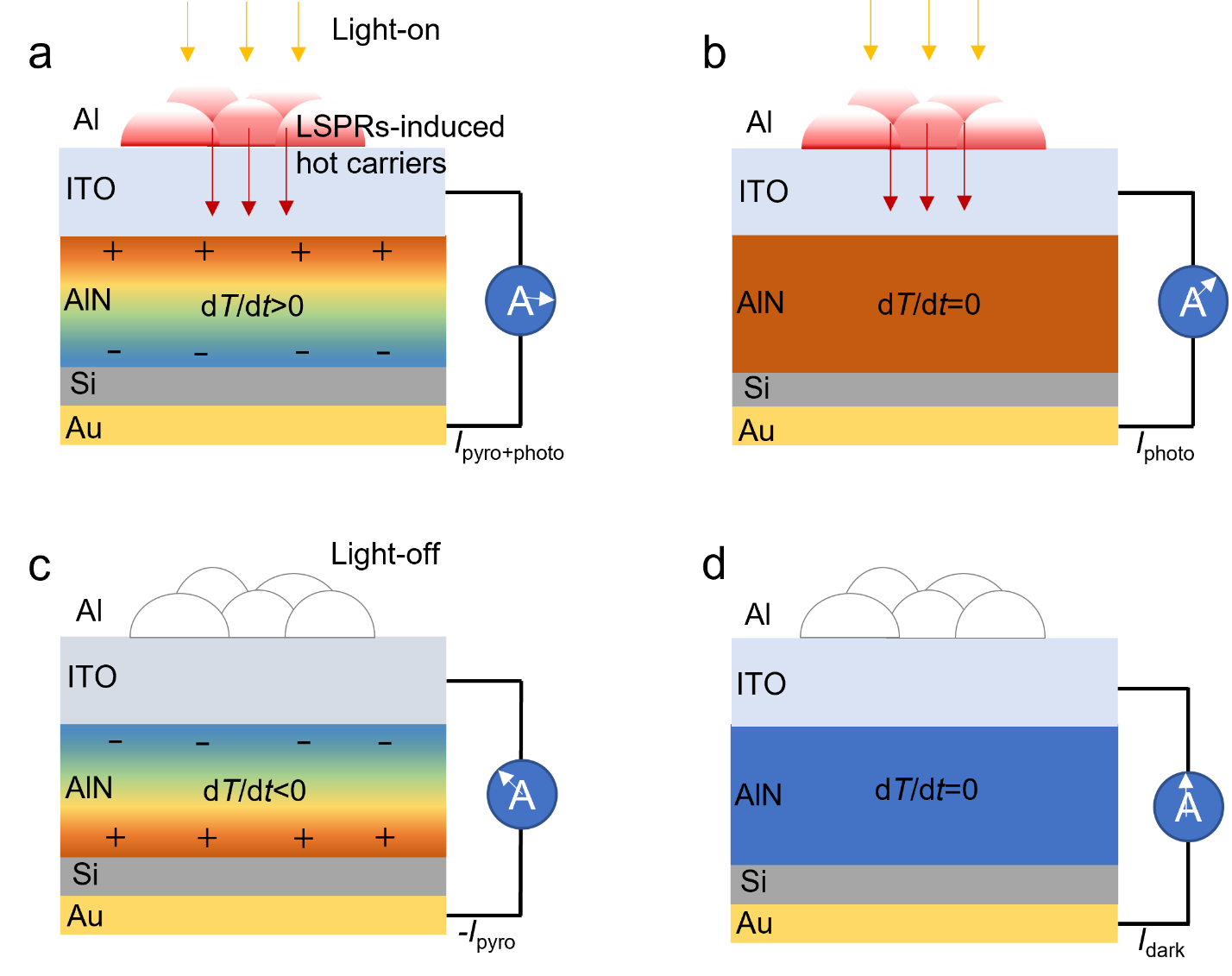


**Fig. S2 Schematic of the operating principle of the proposed pyroelectric detector.** **a** Light-on: The temperature rise of AlN layer induces the *I*_pyro_, while the LSPRs excited by Al particles induce the *I*_photo_. **b** The temperature is stable, and the *I*_pyro_ disappears. **c** Light-off: The *I*_photo_ disappears, while the temperature decrease of AlN layer induces the reverse *I*_pyro_. **d** The temperature is stable, and all the currents disappear.


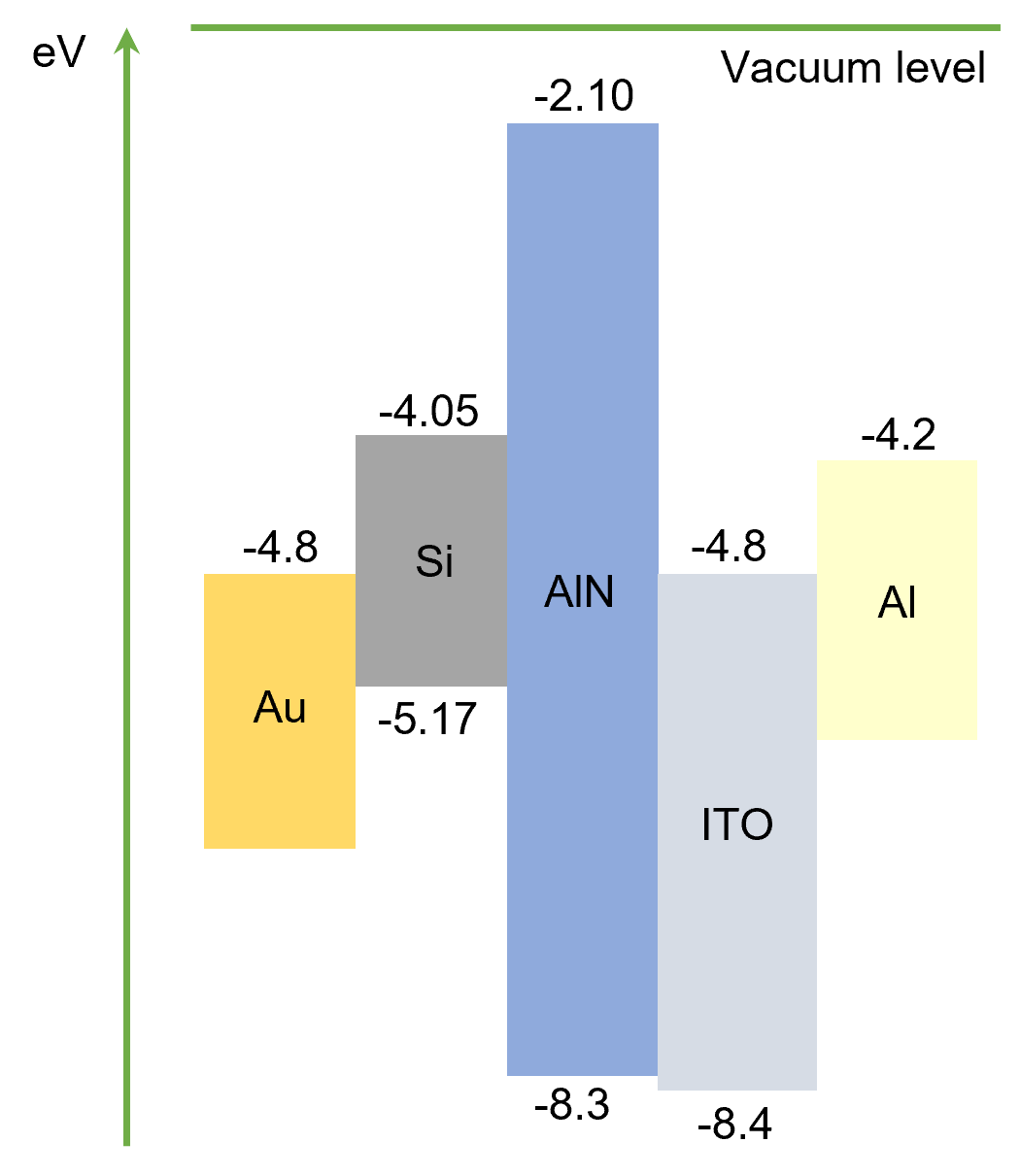


**Fig. S3 Energy band diagram of the proposed pyroelectric detector.** Energies are expressed in electron volts, using the electron energy in vacuum for reference.


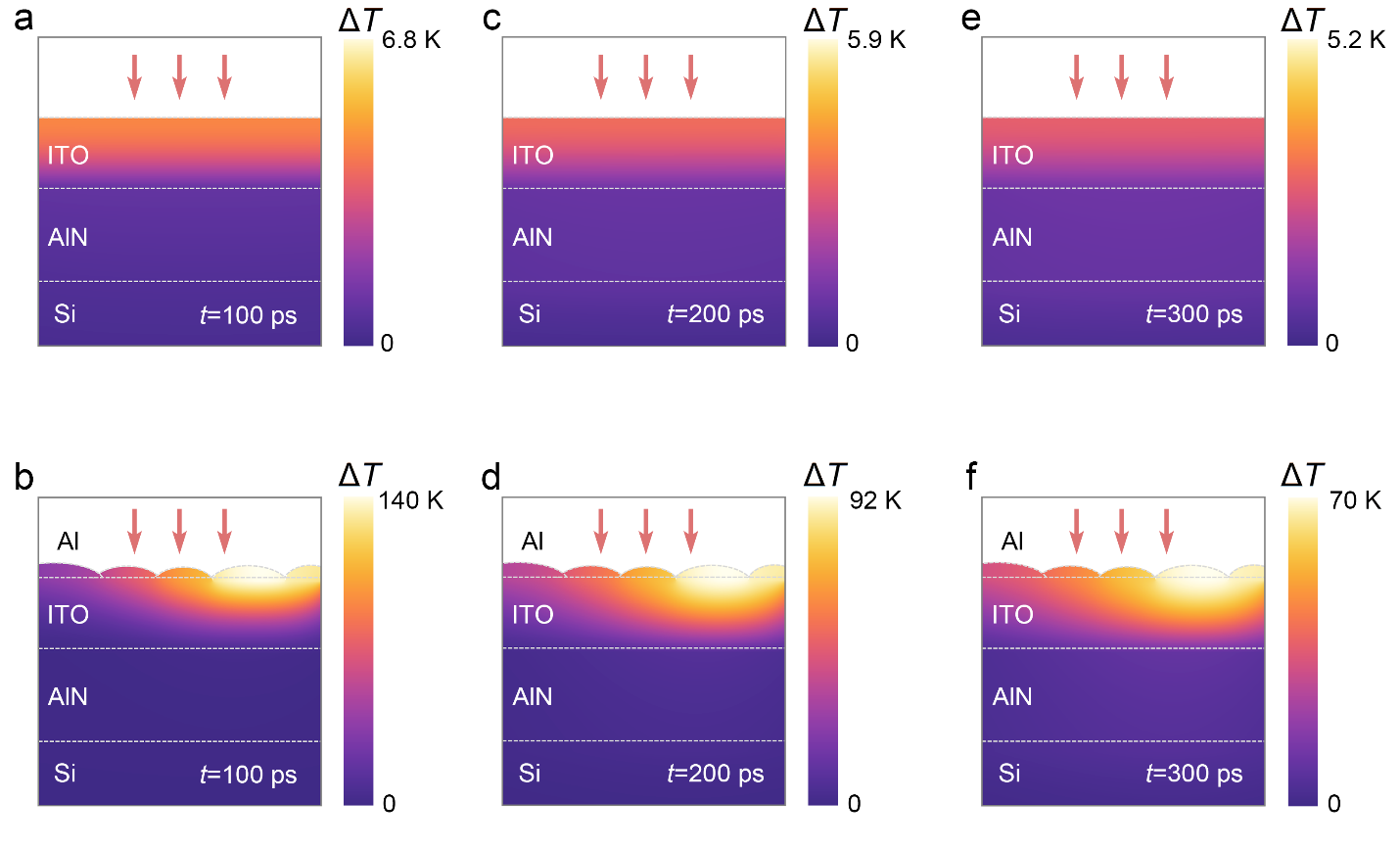


**Fig. S4 Simulated distribution of temperature variation for the control sample and the proposed pyroelectric detector with plasmonic metasurface.** The times of above simulations are **a**, **b** 100 ps, **c**, **d** 200 ps, and **e**, **f** 300 ps. The width of incident light pulse is 200 fs.

**
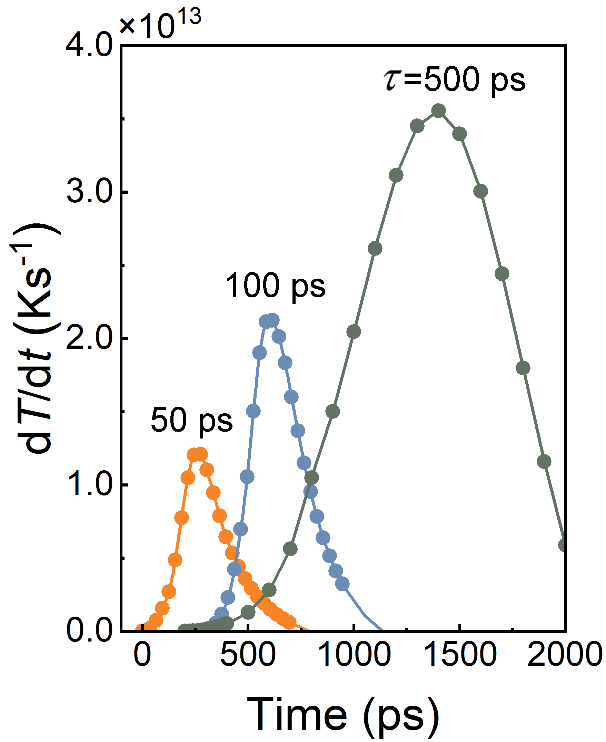
**

**Fig. S5 Simulated d*T*/d*t* of AlN layer induced by different incident light pulses.** The pulse widths *τ* are respectively 50, 100 and 500 ps.

**
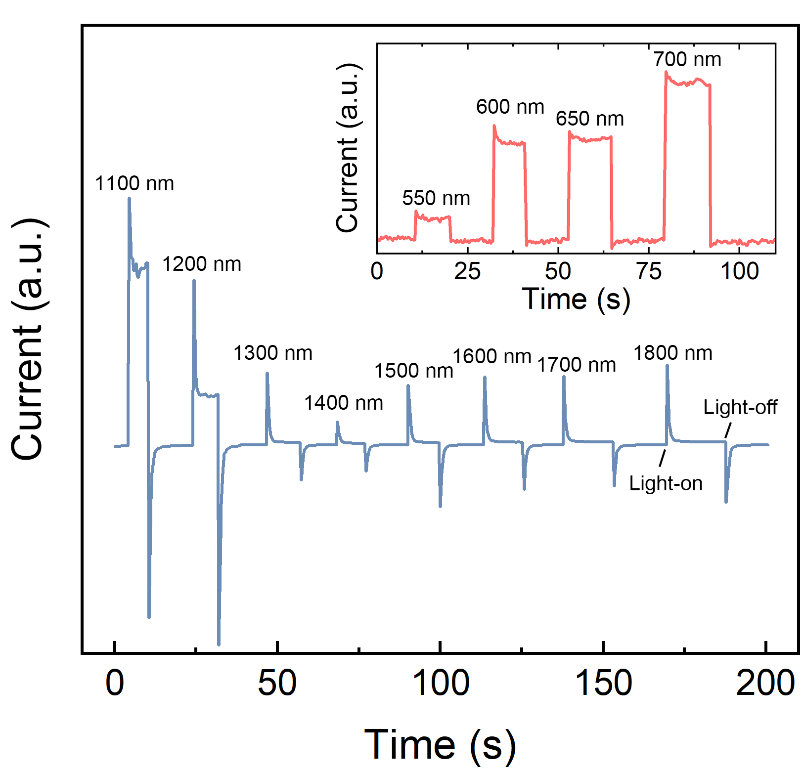
**

**Fig. S6 Switching characteristic profiles of the proposed pyroelectric detector in visible (550−700 nm) and NIR (1100−1800 nm) ranges.**

External quantum efficiency of the PE detector

$\text{R}\text{=}\frac{\text{η}_{\text{e}}\text{q}}{\text{h}\text{n}}$ (S1)

As shown in Eq. S1, the responsivity *R* of a detector can be expressed in terms of its external quantum efficiency (*EQE*) *η*_e_, which is the microscopic characterization of the photoelectric conversion efficiency. Here *q*, *h*, and *ν* are the elementary charge, Planck’s constant, and frequency of incident light, respectively. Figure S7a is the optical power of the incident light used for calculating *R* in the main text, while Fig. S7b exhibits the calculated *EQE* of the proposed pyroelectric detector.


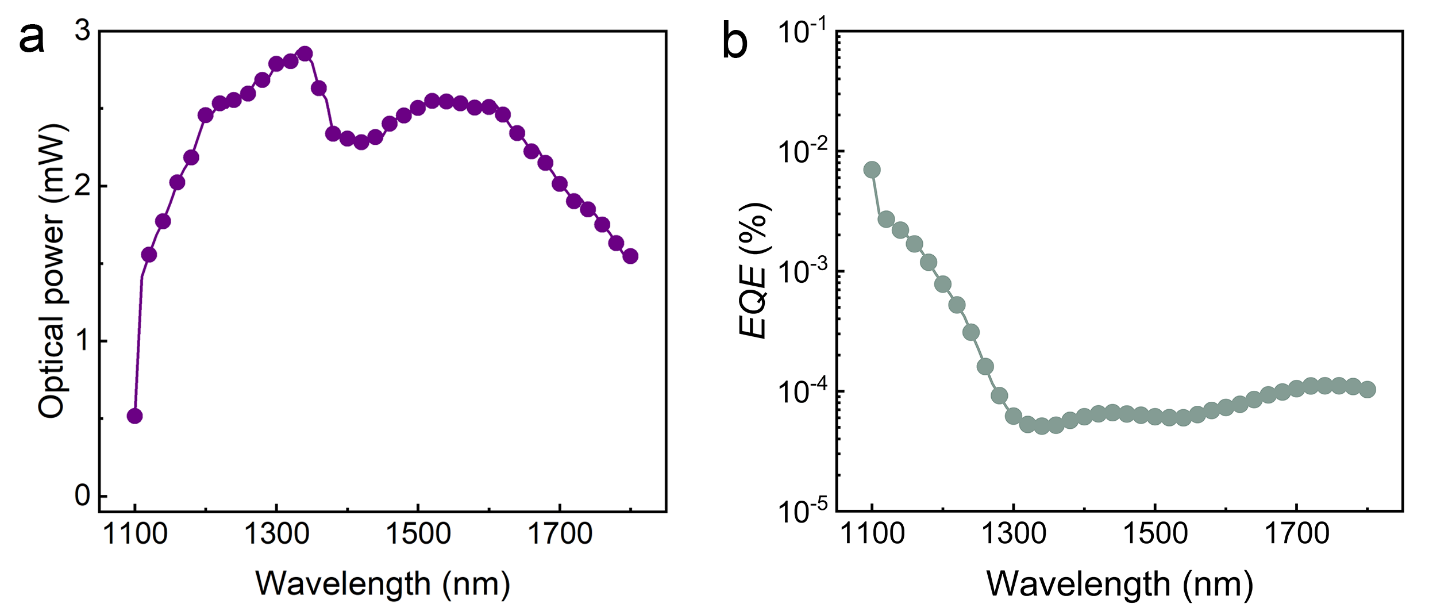


**Fig. S7 a** Optical power of the incident light. **b** External quantum efficiency (*EQE*) of the proposed pyroelectric detector.





**Fig. S8 Response time of proposed detectors at each wavelength.** The red points represent the average values of response times, and the error bars indicate the ranges of response times obtained in repeated measurements.


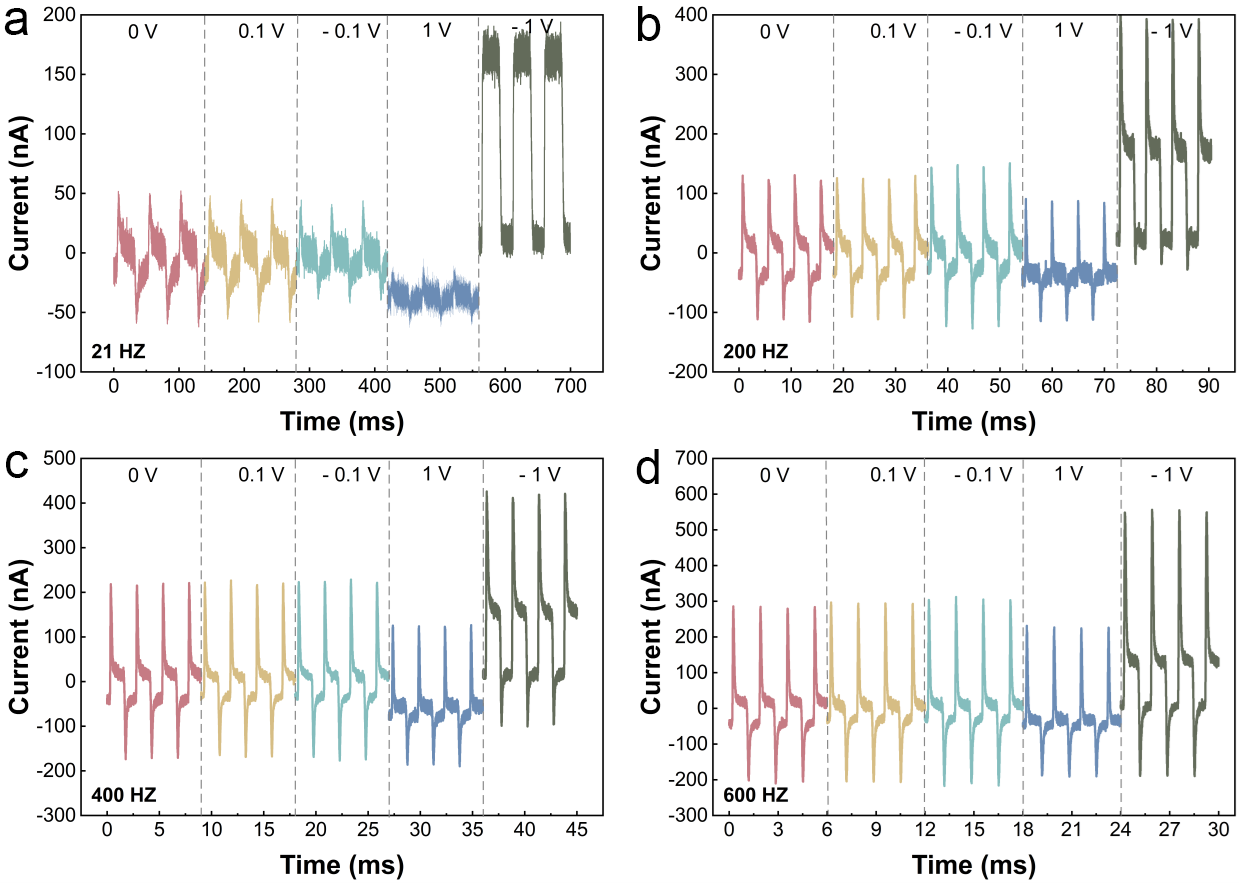


**Fig. S9 Measured response waveforms of the proposed pyroelectric detector under different bias voltages.** The chopper frequencies are **a** 21 Hz, **b** 200 Hz, **c** 400 Hz, and **d** 600 Hz.


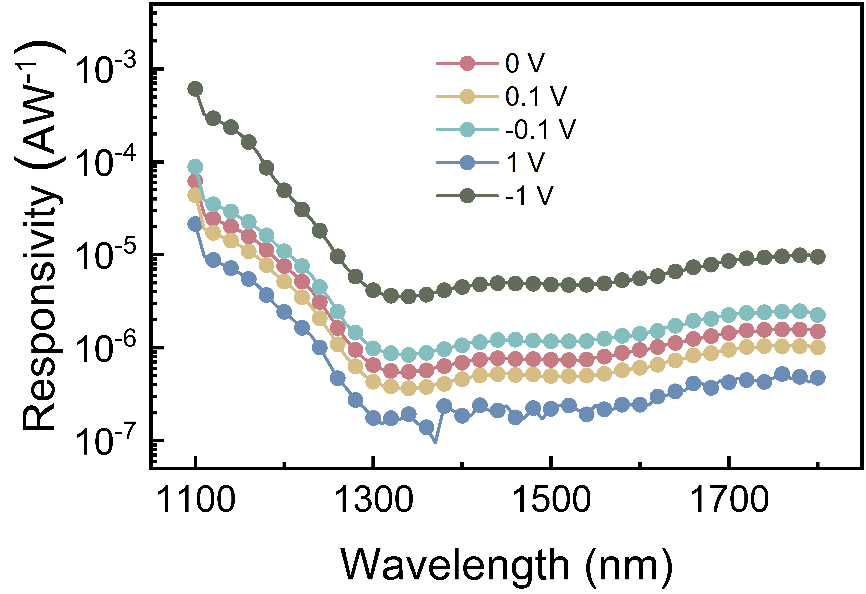


**Fig. S10 Responsivity of the proposed pyroelectric detector obtained with different bias voltages.**


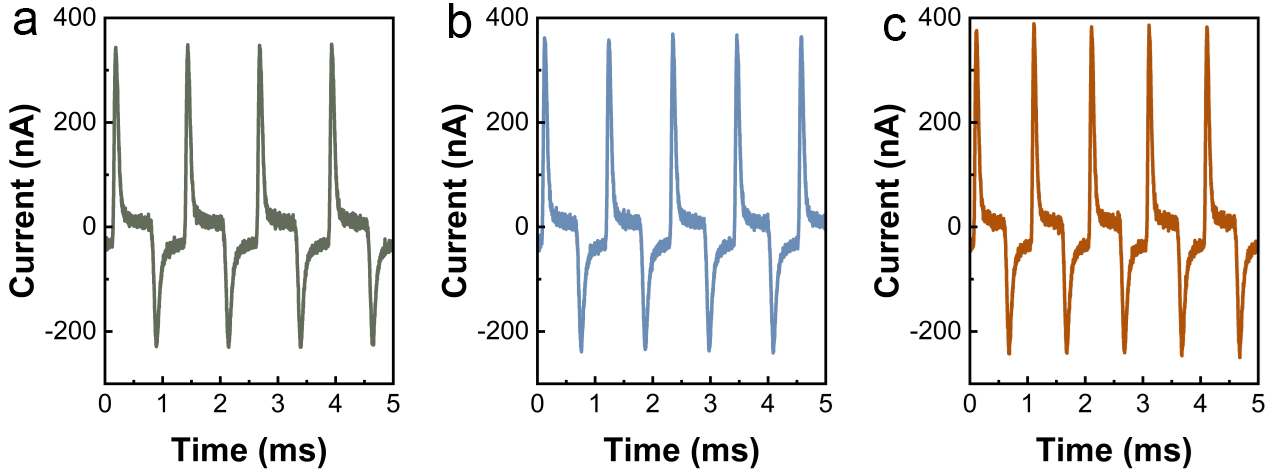


**Fig. S11 Measured response waveforms of the proposed pyroelectric detector at zero bias.** The chopper frequencies are **a** 800 Hz, **b** 900 Hz and **c** 1000 Hz.
